# Is Immediate Recall test scores affected by Anxiety in a memory clinic population? A clinical correlation study

**DOI:** 10.1093/geroni/igaa057.911

**Published:** 2020-12-16

**Authors:** Hamed Khachan, Anil Nair, Fioralba Andrea, Mahak Kanjolia

**Affiliations:** Alzheimer’s Disease Center, Quincy, Massachusetts, United States

## Abstract

Background: It is unknown if anxiety affects performance on immediate recall testing (IR) in memory clinic patients. Method: We performed a retrospective analysis of memory clinic patients in the south shore of Boston from 2010 to 2019. We correlated anxiety screen data (GAD7) to IR scores. Univariate analyses used Spearman correlation. A multivariate regression model analyzed GAD7 to covariates of IR, age, sex, and race. Hypothesis: We hypothesized a positive correlation between anxiety levels scored by GAD7 and IR. Results: 994 patients in the memory clinic between 2010-2020 had analyzable data. Patients were 58.6% female, 84.6 % White. The mean age was 70.1±14.4, IR 6.62 ± 5.4, GAD7 5.5±5.71. On univariate analysis, IR correlated significantly to age (⍴ = 0.08, p = 0.01), gender (⍴ = 0.06, p = 0.046), and race (⍴= - 0.25, p <0.001), but not to GAD7 (⍴=-0.07, p=0.14). The multivariate model confirmed the lack of association of anxiety scores to (□=-0.05, p=0.41) to GAD7 scores. IR task performance was significantly associated only to age (□= -0.04, p=0.03) and gender (□= -1.18, p=0.04) in the regression model. Conclusions: Immediate recall task performance was not significantly affected by anxiety measured by GAD7 scores in a memory clinic population. However, a negative correlation was shown on immediate recall scores in males and older subjects.

